# Effect of elexacaftor-tezacaftor-ivacaftor on nasal potential difference and lung function in Phe508del rats

**DOI:** 10.3389/fphar.2024.1362325

**Published:** 2024-03-13

**Authors:** Nicole Reyne, Patricia Cmielewski, Alexandra McCarron, Ronan Smith, Elena Schneider-Futschik, Nina Eikelis, Piraveen Pirakalathanan, David Parsons, Martin Donnelley

**Affiliations:** ^1^ Robinson Research Institute, University of Adelaide, Adelaide, SA, Australia; ^2^ Adelaide Medical School, University of Adelaide, Adelaide, SA, Australia; ^3^ Respiratory and Sleep Medicine, Women’s and Children’s Hospital, Adelaide, SA, Australia; ^4^ Department of Biochemistry and Pharmacology, School of Biomedical Sciences, Faculty of Medicine, Dentistry and Health Sciences, The University of Melbourne, Australia; ^5^ 4DMedical, Victoria, Australia

**Keywords:** cystic fibrosis, rat model, nasal potential difference, CFTR modulator, lung function, X-ray velocimetry

## Abstract

**Introduction:** *Phe508del* is the most common cystic fibrosis transmembrane conductance regulator (CFTR) gene variant that results in the recessive genetic disorder cystic fibrosis (CF). The recent development of highly effective CFTR modulator therapies has led to significant health improvements in individuals with this mutation. While numerous animal models of CF exist, few have a CFTR mutation that is amenable to the triple combination therapy elexacaftor-tezacaftor-ivacaftor (ETI).

**Methods:** To determine the responsiveness of *Phe508del* rats to ETI, a baseline nasal potential difference was measured. Subsequently, they received ETI daily for 14 days, after which post-treatment nasal potential difference, lung mechanics (via flexiVent) and lung ventilation (via X-ray Velocimetry) were assessed.

**Results:** Chloride ion transport in nasal airways was restored in *Phe508del* rats treated with ETI, but neither lung mechanics nor ventilation were significantly altered.

**Discussion:** These findings validate the usefulness of this rat model for future investigations of modulator therapy in CF.

## Introduction

Cystic fibrosis (CF) is a recessive disorder that is caused by variants in the CF transmembrane conductance regulator (*CFTR*) gene, an ion channel that moves chloride ions across epithelial cells in many organs (Bareil and Bergougnoux, 2020). *CFTR* mutations affect the CFTR protein at different levels, and can alter synthesis, trafficking, maturation, gating and stability. There are more than 2000 identified mutations of the *CFTR* gene, although only ∼300 of these are disease causing (Pereira et al., 2019). Loss of chloride and bicarbonate ion transport in the lung causes airway surface dehydration, allowing thick sticky mucus to accumulate, creating an ideal environment for pathogens to colonise (Bhagirath et al., 2016). The lungs become chronically inflamed and infected, contributing to progressive lung disease, the major cause of mortality and poor quality of life in people with CF.

The *Phe508del* mutation—a deletion of phenylalanine at position 508—is the most common *CFTR* mutation, with ∼80% of people with CF carrying at least one copy. *Phe508del* is a class II *CFTR* mutation, which results in defective CFTR protein folding and maturation, retention in the endoplasmic reticulum, and subsequent protein degradation (Bareil and Bergougnoux, 2020). A class of drugs termed CFTR modulators have been developed to improve the processing and function of CFTR, and consist of 1) correctors, to stabilise the folding of the CFTR protein and limit its degradation, enabling it to be trafficked to the surface (i.e., lumacaftor, tezacaftor and elexacaftor); and 2) potentiators, to improve defective gating, by holding the CFTR channel open long enough for chloride to flow through (i.e., ivacaftor) (Lopes-Pacheco, 2020). To restore chloride transport in people with the *Phe508del* mutations, two steps are required. First, the correction of the processing/trafficking defect with a corrector and second, increasing the channel opening with a potentiator (Haq et al., 2019).

The triple combination CFTR modulator of elexacaftor, tezacaftor, ivacaftor (ETI, marketed as TrikaftaⓇ or KaftrioⓇ) has produced significant health improvements in people with CF that have one or two copies of the *Phe508del* variant (Heijerman et al., 2019; Middleton et al., 2019). ETI has shown substantial improvements in lung function, increase in body mass index and a reduction in the number and frequency of pulmonary exacerbations (Heijerman et al., 2019). Understanding the long-term safety and effectiveness of triple combination modulators is a focus of groups such as the Cystic Fibrosis Foundation (Nichols et al., 2021).

Recently a *Phe508del* CF rat model has been characterised (McCarron et al., 2020), and shown to display an array of CF pathologies that are significantly different from their wildtype counterparts. These include reduced survival, intestinal obstructions, defects in the male reproductive, altered nasal bioelectrical profile, and subtle differences in lung function. In the present study we investigate the responsiveness of *Phe508del* rats to ETI, examining nasal potential differences (PD) and lung function (flexiVent/X-Ray Velocimetry).

## Materials and methods

### Animals

All animal procedures were approved by the University of Adelaide Animal Ethics Committee under application M-2021-032 and adhered to the ARRIVE guidelines (Percie du Sert et al., 2020). Female *Phe508del* rats (McCarron et al., 2020) (n = 9) were used, and were 34–40 days old at the start of the 2-week ETI treatment. Wildtype (n = 6; average 62 days old) and untreated *Phe508del* (n = 9; average 70 days old) female rats were included as controls for the lung function and XV imaging. All rats were maintained in conventional cages with a 12-h light/dark cycle. Food and water were provided *ab libitum*, with all rats receiving a 50:50 mix of normal and high fat (10%) rodent chow. CF rats received water containing 4.5% ColonLytley (Dendy Pharmaceuticals, Australia) to minimise gut obstructions.

### Treatment with ETI

Nine *Phe508del* rats received ETI twice daily for 14 days. Each modulator was dissolved in dimethyl sulfoxide (DMSO) and mixed into peanut butter at the correct dose for the weight of the rat. The morning dose consisted of ivacaftor (25 mg/kg/day) and the evening dose ivacaftor (25 mg/kg/day), elexacaftor (6.7 mg/kg/day) and tezacaftor (3.5 mg/kg/day).

### Nasal potential difference

Ion channel function was measured in the *Phe50del* rat treatment group, before ETI dosing began and again after 14 days of ETI, using the nasal potential difference (PD) method as previously described (Reyne et al., 2021). Rats were anaesthetised with 60–75 mg/kg of a mixture of ketamine (Ceva, Australia) and medetomidine 0.4 mg/kg (Ilium, Australia), delivered by intraperitoneal injection.

Briefly, nasal PD values were recorded using an optically-isolated high impedance mV meter (ISOMIL, World Precision Instruments), connected to an eight channel PowerLab data acquisition unit combined with LabChart software (Version 8, ADIntruments). The nasal cannula was inserted 4–7 mm into the nasal cavity and the reference electrode inserted subcutaneously into the abdomen, and connected to calomel electrodes (Hg_2_Cl_2_ in 3 M KCl, Cole-Parmer Instruments, United States).

The nasal cavity was perfused at a rate of 10 μL/min with the following solutions: 1) normal Krebs-Ringer buffer (KRB), 2) KRB containing 100 μM amiloride, 3) low chloride KRB with 100 μM of amiloride, and 4) low chloride KRB containing 100 μM of amiloride and 100 μM of isoproterenol. Each solution was perfused for 10–15 min until a plateau of 1–2 min was obtained. After PD measurements were completed, anaesthesia was reversed with 1 mg/kg atipamezole (Jurox, Australia) and returned to their home cages.

#### Lung function—X-ray velocimetry (XV)

The ETI treated *Phe508del* rats, control untreated *Phe508del* rats, and WT rats were anaesthetised as above. Once anaesthetised, the rats were prepared by performing a tracheostomy and cannulation with a shortened endotracheal tube (ET; 14 Ga BD Insyte plastic cannula bevel cut to 15 mm length), and then positioned into a custom 3D printed holder. XV imaging was performed with a Permetium preclinical scanner (4DMedical, Australia), consisting of a Rigaku MM09 X-ray source (40 kV, 20 mA) with Molybdenum anode and an 80 μm spot size, combined with a Varex 2020DX flat-panel detector. A 1700 mm sample to detector distance was utilised to facilitate propagation-based phase contrast X-ray imaging. For imaging, rats were placed onto the translation/rotation stage of the Permetium. Animals were connected to a small animal ventilator (Accuvent 200, 4DMedical, Australia) set to a peak inspiratory pressure of 14 cmH_2_O, positive end-expiratory pressure of 2 cmH_2_O, and ventilated at 93.75 breaths/min (192 m inspiration and 448 m expiration; I:E ratio of 3:7). A single 4D XV scan was acquired at a framerate of 15.63 Hz, with 10 images taken per breath and 600 projections per phase-point. XV scan data was sent to 4DMedical for analysis for processing to provide measures of the mean specific ventilation, ventilation defect percentage, and ventilation heterogeneity.

#### Lung mechanics - flexiVent

Following XV imaging, lung function assessments were performed using a flexiVent FX small animal ventilator fitted with a FX4 rat module (SCIREQ, Montreal, Canada). The 14 Ga cannula was connected to the flexiVent and ventilatory parameters set at respiratory rate 90 breaths/min, tidal volume 10 mL/kg and positive end expiratory pressure (PEEP) of 3 cmH_2_O. Lung mechanics measurements were made using the Scireq automated algorithms configured in a rat mechanics scan script that was repeated three times.

A deep inflation manoeuvre was applied to the lungs to maximally inflate the lungs to a pressure of 30 cmH_2_O over a period of 3 s to recruit closed lung areas, standardise lung volume history, and calculate the inspiratory capacity (IC). A SnapShot-90 perturbation, performed at a single frequency to match the rat respiratory rate and tidal volume was then used to fit the single-compartment model and measure the respiratory system resistance (R_rs_), compliance (C_rs_), and elastance (E_rs_). A Quick-Prime 3 forced-oscillation perturbation using a range of frequencies above and below the respiratory rate was then applied, with the software fitting the constant-phase model of respiratory system impedance (Z_rs_) to calculate Newtonian resistance (R_n_), tissue damping (G), and tissue elastance (H). The parameter Eta called hysteresivity—a measure of heterogeneity—was calculated by dividing G by H. Maximal volume-controlled stepwise pressure-volume (PVs-V) loops were generated and the Salazar-Knowles equation automatically fitted to the expiratory data to obtain the maximal lung capacity (A) curvature of the deflating PV loop (K), and quasi-static compliance (C_st_). The PV loop area was also calculated. For each parameter, an average of three measurements was calculated per rat. Data was excluded if the coefficient of determination—a measure of model fit—was less than 0.9 for each model. IC was normalised to body weight.

## Statistics

Statistical analyses were performed in R version 4.2.0 (R Core Team, 2019), and statistical significance was set at *p* = 0.05. Sample characteristics were assessed by one-way ANOVA followed by Tukey’s Test, as appropriate, to determine whether the groups differed in age or weight. Nasal PD data was analysed using paired *t*-test to compare the PD measurements for each rat prior to ETI and post-ETI-treatment. For flexiVent and XV parameters a linear regression was performed using the “lm” function, with fixed effects of genotype and weight. Pairwise comparisons for the fitted model were performed using the “emmeans'' package (Pinheiro et al., 2007).

## Results

### Animals

A total of nine *Phe508del* rats received ETI, however due to errors in the post-treatment nasal PD assessment, two were removed from the study. To be able to compare the effects of ETI on lung function, six wildtype and nine *Phe508del* rats were included as controls (no treatment) for the flexiVent and XV assessments. There was no significant difference in the age of the rats, however the *Phe508del* + ETI weighed significantly less than the wildtype and *Phe508del* rats (*p* = 0.049 and *p* = 0.034, respectively).

### Nasal potential difference demonstrates restored chloride ion transport following ETI treatment

**FIGURE 1:**
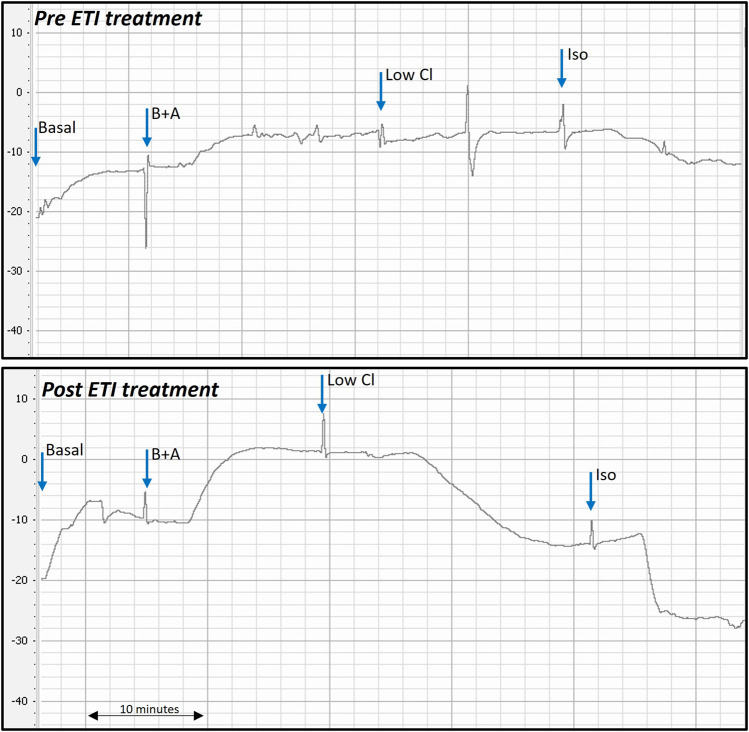
Representative nasal PD trace of a *Phe508del* rat prior to and after ETI treatment. Treated *Phe508del* rats all demonstrated correction of the bioelectric defect. Correction of the bioelectric defect is demonstrated by the more negative PD response to the low chloride and isoproterenol solutions, compared to little/no response pre ETI treatment. (B + A: basal + amiloride, Low Cl: low chloride + amiloride, Iso: isoproterenol + low chloride + amiloride. Blue arrows show when each solution was changed).

**FIGURE 2:**
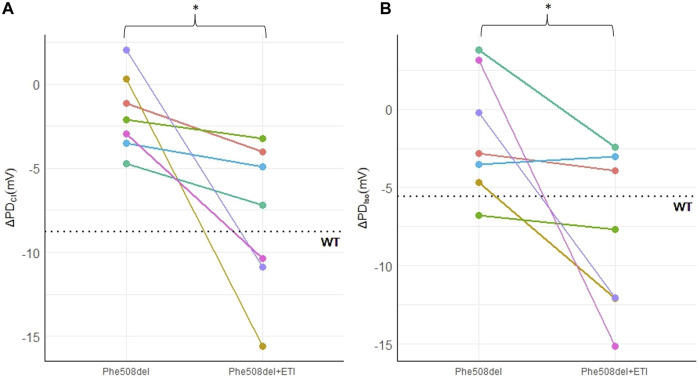
ETI restores nasal chloride ion transport in a *Phe508del* rat model. Nasal PD measurements in *Phe508del* and *Phe508del* + ETI rats for **(A)** ΔPD_Cl_ and **(B)** ΔPD_Iso_. Dotted line indicates the wildtype average (*p < 0.05, paired *t*-test, n = 7). Data represented as each individual rat pre and post ETI treatment.

Representative PD traces from the same *Phe508del* rat pre- and post-ETI, shows correction of the bioelectrical defect in the *Phe508del* + ETI rat after 14 days of ETI treatment (Figure 1). After 14 days of ETI treatment *Phe508del* rats (n = 7) showed a more negative delta-low-chloride response (−8.04 ± 1.7 mV), which was significantly different from pre-treatment (−1.73 ± 0.88 mV, *p* = 0.032) (Figure 2A). The isoproterenol response produced a larger hyperpolarization in treated *Phe508del* + ETI rats (−8.06 ± 1.9 mV) compared to baseline (−1.59 ± 1.5 mV) (*p* = 0.046), indicating CFTR-mediated chloride transport is corrected (Figure 2B). The basal KRB and amiloride responses in the treated *Phe508del* + ETI rats were not significantly different from baseline.

### Lung function results showed that there was no statistically significant difference between wildtype and Phe508del treated rats after ETI treatment

**FIGURE 3:**
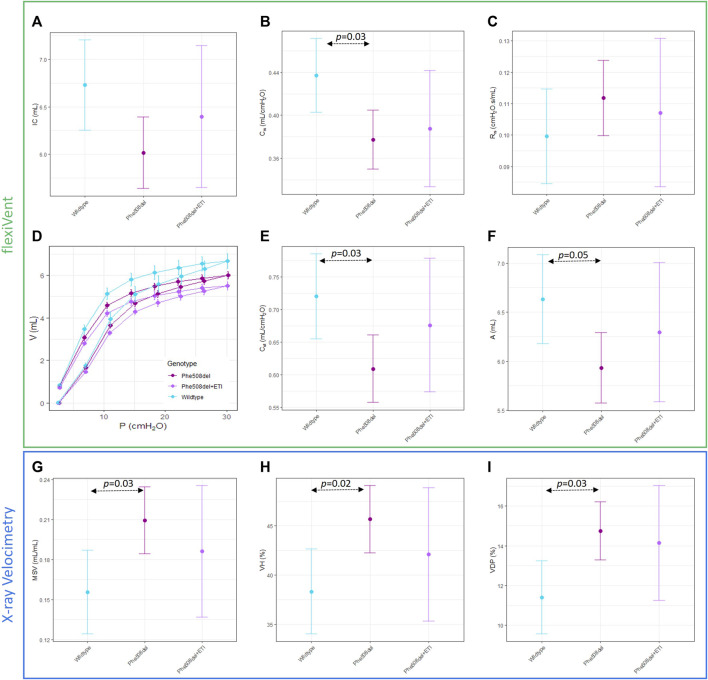
Overall mechanics of the respiratory system are not significantly altered with ETI. Single compartment analysis of respiratory system using flexiVent forced oscillation technique in wildtype, *Phe508del* and *Phe508del* + ETI. **(A)** Inspiratory capacity (IC), **(B)** compliance (C_rs_), and **(C)** Resistance (R_rs_) Baseline pressure volume loops were generated using a volume-controlled stepwise manoeuvre for each genotype. **(D)** Raw pressure volume loops, **(E)** static compliance, and **(F)** PV loop area. X-ray Velocimetry parameters were also not significantly altered by ETI treatment, **(G)** mean specific ventilation (MSV), **(H)** ventilation heterogeneity (VH), and **(I)** ventilation defect percentage (VDP). (n = 6–9/treatment, bars show estimated means and 95% CIs).

Significant differences between untreated *Phe508del* and wildtype rats were found in some XV (MSV, VDP, VH) and flexiVent (compliance (Crs), static compliance (Cst), PV loop area) parameters (Figures 3A–F). No significant differences were found between ETI treated *Phe508del* and wildtype rats in these parameters. No significant differences were found between any groups in the other XV and flexiVent parameters (Figure 3). Representative ventilation maps and ventilation histograms of a rat from each group are shown in the Supplementary Material.

## Discussion

This study presents evidence that the respiratory epithelium in *Phe508del* CF rats responds to ETI treatment. Nasal PD assessment demonstrated an *in vivo* restoration for the first time of nasal epithelial CFTR ion transport after 2 weeks of ETI administration to *Phe508del* rats.

There was a significant improvement in both the chloride and isoproterenol response in *Phe508del* rats that received ETI. Treated *Phe508del* rats showed a mean of 36.5% restoration in PD towards the wildtype level for the chloride response. The isoproterenol response was a mean of 83.2% towards the wildtype level in those *Phe508del* rats that received ETI. Together these results indicate that CFTR-mediated chloride transport in the nasal epithelium is corrected in *Phe508del* rats that received ETI.

Lung function and XV scan assessment revealed that there was not a significant difference between the ETI treated *Phe508del* and untreated *Phe508del* rats, although for all parameters the ETI treated group was shifted towards wildtype. This could be because the control *Phe508del* rats only exhibited small differences in lung function compared to wildtype rats, in contrast to humans where the difference between CF and normal is often very large. It is possible that a larger cohort of rats may be necessary to observe a statistically significant effect, or a longer period of treatment with ETI may be needed to drive the *Phe508del* lung phenotype further towards the wildtype animals.

Previous research has demonstrated the efficacy of ivacaftor in rat and ferret models of CF with the Gly551Asp (G551D) mutation (Sun et al., 2019; Birket et al., 2020), although this mutation is less prevalent (2.5%–5% occurrence) in the CF population than the *Phe508del* mutation. Treating pregnant G551D ferrets with ivacaftor prevents CF neonatal mortality associated with gut obstructions, produces partial recovery of pancreatic function, slows lung disease progression and improves weight gain (Sun et al., 2019). The G551D rat model is a humanised model, expressing *hCFTR* with the G551D mutation, and having the human CFTR sequence in the genome may be more relevant for testing modulator therapy in relation to predicting outcomes in humans. In their model, ivacaftor restored transepithelial nasal PD and mucus defects were normalised, however they did not assess how lung function was affected (Birket et al., 2020). In another *Phe508del* rat model, the effect of VX-770 (ivacaftor) and VX-809 (lumacaftor - a corrector) alone or in combination (Orkambi) was investigated by measuring short-circuit current in primary nasal cells (Dreano et al., 2019). No effect was found when ivacaftor or lumacaftor were applied alone, but the combination improved CFTR-dependent chloride transport in *Phe508del* cells. However, that study did not assess the effect of modulator therapy *in vivo*. Given the higher occurrence of the *Phe508del* mutation, the ability to utilise ETI in the rat animal model may provide useful therapeutic understanding and development capability due to its wider applicability in CF.

Looking ahead, the establishment of an ETI- responsive rat model should facilitate investigations into the long-term effects of such modulator treatments. Of particular value is the exploration of the impact of ETI during pregnancy, a dimension that is gaining interest due to the increasing number of females with CF who now bear children while receiving modulator therapy. Studies looking at the safety of drugs on developing foetuses (e.g., evidence of cataracts), and the drug transfer to the foetus via placenta and lactation, are all important areas that could be performed in a rat model (Jain et al., 2022). The potential for utilising CFTR modulators in animal models offers a platform to effectively study therapeutic windows and the impact of disease progression, as well as biodistribution, drug metabolism, drug half-life and drug toxicity (Sun et al., 2019; Grubb and Livraghi-Butrico, 2022).

Another emerging area of interest is the impact of modulator therapy on bacterial infections. Some clinical studies indicate a decline in the bacterial load during the first year of single modulator therapy, followed by a resurgence in the second year (Hisert et al., 2017), while other studies show a persistent reduction in bacterial load following single modulator initiation (Frost et al., 2019). In the era of modulator therapy, it still remains uncertain how pathogens, particularly *Pseudomonas aeruginosa*, will adapt and change with ETI treatment (Greenwald and Wolfgang, 2022).

While short term studies in people with CF have shown positive outcomes it is crucial to investigate the sustained effect and safety of these modulator therapies over time (Heijerman et al., 2019; Middleton et al., 2019). Questions about potential side effects with prolonged use, and the impact of disease progression are areas that are currently being investigated (Nichols et al., 2021). As with any new treatment, the long term effects of triple combination modulators are an area of ongoing research. The availability of the *Phe508del* rat model provides a valuable platform for conducting these investigations.

In summary, our results demonstrate successful restoration of CFTR chloride ion transport using triple modulator therapy in *Phe508del* rats for the first time, and provide a new capability for research into the effects of ETI on CF lung health.

## Data Availability

The original contributions presented in the study are included in the article/Supplementary Material, further inquiries can be directed to the corresponding author.
