# Corrigendum: Effect of elexacaftor-tezacaftor-ivacaftor on nasal potential difference and lung function in Phe508del rats

**DOI:** 10.3389/fphar.2024.1428213

**Published:** 2024-05-28

**Authors:** Nicole Reyne, Patricia Cmielewski, Alexandra McCarron, Ronan Smith, Elena Schneider-Futschik, Nina Eikelis, Piraveen Pirakalathanan, David Parsons, Martin Donnelley

**Affiliations:** ^1^ Robinson Research Institute, University of Adelaide, Adelaide, SA, Australia; ^2^ Adelaide Medical School, University of Adelaide, Adelaide, SA, Australia; ^3^ Respiratory and Sleep Medicine, Women’s and Children’s Hospital, Adelaide, SA, Australia; ^4^ Department of Biochemistry and Pharmacology, School of Biomedical Sciences, Faculty of Medicine, Dentistry and Health Sciences, The University of Melbourne, Parkville, VIC, Australia; ^5^ 4DMedical, Carlton, VIC, Australia

**Keywords:** cystic fibrosis, rat model, nasal potential difference, CFTR modulator, lung function, X-ray velocimetry

In the published article, there was an error in the author list, and author Elena Schneider-Futschik was erroneously excluded. Consequently, the affiliation of the author Elena Schneider-Futschik is now added to the published article as **affiliation 4** “Department of Biochemistry and Pharmacology, School of Biomedical Sciences, Faculty of Medicine, Dentistry and Health Sciences, The University of Melbourne, Australia”. The original **affiliation 4** (4DMedical, Victoria, Australia) is now renumbered to **affiliation 5**

The corrected author list appears below.

Nicole Reyne, Patricia Cmielewski, Alexandra McCarron, Ronan Smith, Elena Schneider-Futschik, Nina Eikelis, Piraveen Pirakalathanan, David Parsons and Martin Donnelley

The **Author Contributions** section is also updated to reflect the addition of the author Elena Schneider-Futschik. The correct **Author Contributions** section appears below.

