# Supplementary figures and images for: Effect of elexacaftor-tezacaftor-ivacaftor on nasal potential difference and lung function in Phe508del rats
Source: Front Pharmacol. 2024 Mar 13;15:1362325. doi: 10.3389/fphar.2024.1362325 (PMC10965794; doi:10.3389/fphar.2024.1362325)

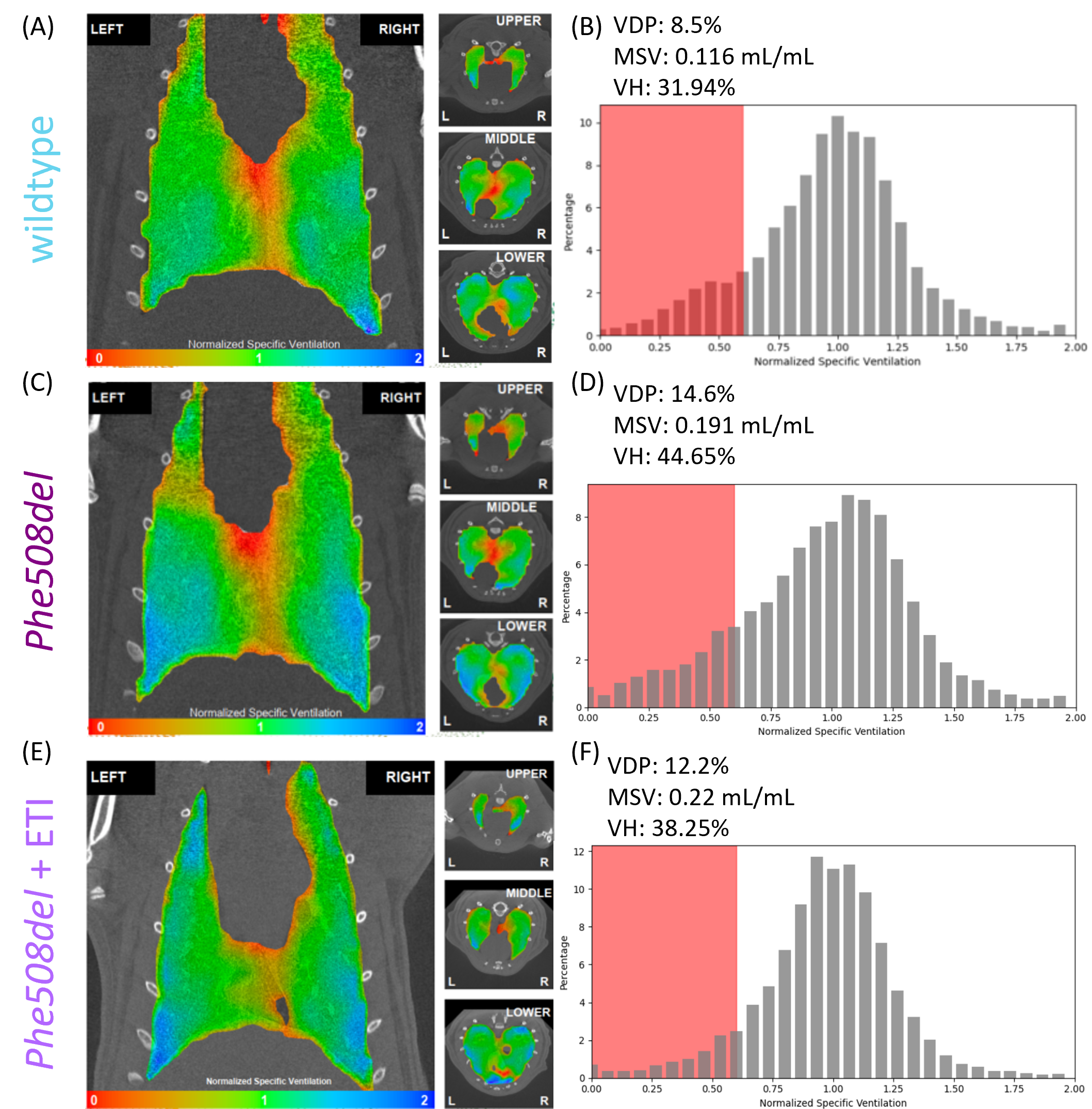

Supplement: Supplementary file 1 [file Image1.PNG]
